# Long noncoding RNA DANCR promotes invasion of prostate cancer through epigenetically silencing expression of TIMP2/3

**DOI:** 10.18632/oncotarget.9350

**Published:** 2016-05-13

**Authors:** Jing Jia, Feng Li, Xiao-Shuang Tang, Shan Xu, Yang Gao, Qi Shi, Wenhuan Guo, Xinyang Wang, Dalin He, Peng Guo

**Affiliations:** ^1^ Department of Urology, The First Affiliated Hospital of Xi'an Jiaotong University, Xi'an, Shaanxi, China; ^2^ Oncology Research Lab, Key Laboratory of Environment and Genes Related to Diseases, Ministry of Education, Xi'an, Shaanxi, China; ^3^ Department of Urology, University of Pittsburgh School of Medicine, Pittsburgh, Pennsylvania, USA

**Keywords:** DANCR, prostate cancer, TIMP2, TIMP3, EZH2

## Abstract

LncRNA DANCR suppresses differentiation of epithelial cells, however, its function in prostate cancer development is still unknown. In the present study, we found the expression of DANCR increases in prostate cancer tissues and cells compared to normal prostate tissues and cells, moreover, DANCR promotes invasion and migration of prostate cancer cells *in vitro* and metastasis of tumor xenografts in nude mice. Mechanistically, we found that TIMP2/3, which are critical metastasis inhibitor of prostate cancer, were down-regulated by DANCR synergistically with EZH2 through epigenetically silencing their promoter by chromatin immunoprecipitation assay. In addition, we further investigated whether DANCR is regulated by the differentiation-promoting androgen-androgen receptor (AR) pathway and found that DANCR expression is repressed by androgen-AR; furthermore, DANCR impedes the upregulation of TIMP2/3 and the suppression of invasion and migration by androgen-AR. On the other hand, interestingly, we found that in prostate cancer cells DANCR knockdown decreased the promotion of invasion and migration by the treatment of enzalutamide, which is an AR inhibitor. In summary, our results indicate that DANCR promotes prostate cancer invasion and metastasis through repressing the expression of TIMP2/3, and suggest that DANCR could be a potential target for preventing prostate cancer metastasis, and knockdown DANCR may lessen the potential side effect of AR inhibitor.

## INTRODUCTION

Prostate cancer is one of the most prevalent solid tumor and a leading cause of cancer-related deaths among males in the U.S., with an estimated 29,720 deaths in 2013 [1]. Androgen receptor (AR) plays a critical role in the development of prostate cancer, and androgen deprivation therapy (ADT) is the first line therapy for most first time diagnostic prostate cancer patients [2–3]. However, despite initial response rates of 80–90%, patients will progress to castration-resistant prostate cancer (CRPC) and even metastatic prostate cancer [4–3]. In prostate cancer, the development of metastasis essentially means the patient is incurable and treatments so far have had only modest effects on survival [4]. Therefore, developing novel approaches to prevent the metastasis of prostate cancer is urgently needed.

Long Noncoding RNAs (lncRNAs) are a class of noncoding RNAs which are longer than 200 nucleotides without evident protein coding function [5], and they express at much lower levels and with much higher cell- type specificity than protein coding mRNAs [6]. Within the last few years, thousands of lncRNAs have been identified and their function in biological processes begun to be understood, for example, lncRNAs often regulate chromatin states by association with chromatin-modifying proteins, such as the polycomb repressive complexes (PRC2) [7–5]. Recently, many studies have shown that lncRNAs frequently dysregulate in various cancers and have multiple functions in a wide range of biological processes, such as the cell proliferation, cell apoptosis, cell cycle arrest and cell migration and invasion [8]. A plenty of reports have demonstrated that lncRNAs function as crucial regulators in prostate cancer development and progression in recent years. For instance, the antisense lncRNA PCA3 is the most prominent and clinically relevant RNA biomarker in prostate cancer, which is overexpressed in > 95% of primary tumors [9]. Prostate cancer noncoding RNA 1 (PRNCR1; PCAT- 8), which was identified to be upregulated in aggressive prostate tumors, promotes AR methylation at K 349 and results in ligand-independent activation of AR signaling and cell proliferation [10]. PCAT-18 was recently suggested as a biomarker for metastatic prostate cancer, and knockdown of PCAT-18 decreased cell proliferation, migration, and invasion but also triggered apoptosis by promoting caspase activity [11]. Though the overall pathophysiological function of lncRNAs in prostate cancer remains to be unknown by now, previous studies strongly suggested that lncRNAs could be potential therapeutic targets in prostate cancer.

Given the importance of lncRNAs in prostate cancer, in the present study, we investigated the lncRNA DANCR (Differentiation Antagonizing Non-Protein Coding RNA), which suppresses the differentiation of epidermal cells [12] and promotes the stemness features of hepatocellular carcinoma cells [13]. However, the biological functions and significance of DANCR in other tumors including prostate cancer have not been established yet. In this study, we detected the expression of DANCR in prostate cancer tissues and cell lines, and whether DANCR regulated the migration and invasion of prostate cancer *in vitro* and *in vivo*. Moreover, we determined whether DNACR regulated cell invasion synergistically with EZH2 and whether DANCR affected the effect of androgen-AR signaling and enzalutamide, an inhibitor of AR, on migration and invasion of prostate cancer cells. Finally we found that DANCR could be a potential target for preventing the metastasis of prostate cancer.

## RESULTS

### Expression of DANCR increases in human prostate cancer tissues and cell lines

**Figure 1:**
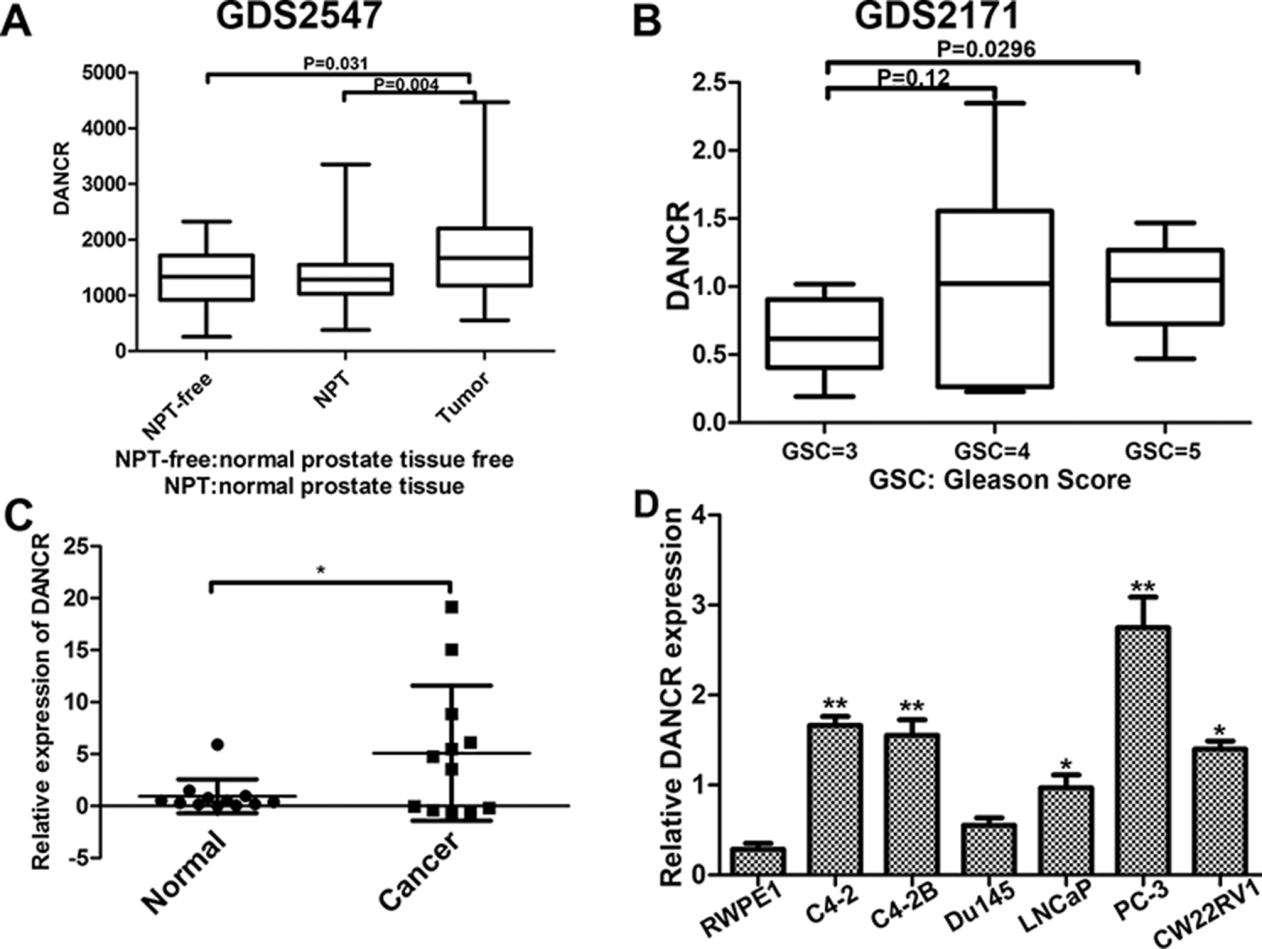
Expression of DANCR increases in human prostate cancer tissues and cell lines (**A**) DANCR expresses higher in prostate cancer tissues than in normal controls. NPT-free: Normal prostate tissue free of any pathological alteration (*n* = 17); NPT: Normal prostate tissue adjacent to tumor (*n* = 58); Tumor: prostate cancer tissues (*n* = 64). Data from GEO profiles. (**B**) Relative expression of DANCR in patients with different Gleason Scores. GSC3: *n* = 10; GSC4: *n* = 12; GSC5: *n* = 8. Data from GEO profiles. (**C**) Human prostate tissues with Gleason score 7 or above were acquired (*n* = 12). Epithelial cells were laser-capture micro-dissected and used for real-time PCR analysis. DANCR mRNA levels were quantified and normalized to GAPDH. All data represent matched pairs of cancer and normal adjacent tissue. (**D**) Relative expression of DANCR in prostate cancer cell lines in comparison with an immortalized normal prostate epithelial cell line. DANCR was examined by RT-qPCR and normalized to GAPDH expression. *p < 0.05.

From the Oncomine database, we found that lncRNA DANCR is up-regulated in prostate cancer. To determine the expression of DANCR in prostate cancer, we first searched multiple microarray data in the GEO database. As shown in Figure 1A, DANCR mRNA levels were significantly upregulated in human prostate cancer tissues compared with normal prostate tissues free of any pathological alteration or normal prostate tissues adjacent to tumor (GSE2547, http://www.ncbi.nlm.nih.gov/geo/). Furthermore, elevated expression of DANCR was associated with high-grade tumors (Gleason score 5 samples versus Gleason score 3 samples) (Figure 1B, GSE2171, http://www.ncbi.nlm.nih.gov/geo/). To confirm the result from the GEO database analysis, we detected the expression of DANCR by RT-qPCR in clinically collected human prostate adenocarcinoma specimens and their paired adjacent normal tissues using laser capture micro-detection, and we found that DANCR level in prostate cancer specimens are much higher than that in adjacent normal tissues (Figure 1C). These results are consistent with a previous report that DANCR expression is significantly higher in prostate cancer samples (*n* = 150) than that in normal prostate samples (*n* = 29) [14]. In addition, we detected the expression level of DANCR in prostate cancer cell lines, and consistently we found that DANCR is up-regulated in prostate cancer cell lines compared with RWPE1, which is an immortalized normal prostate epithelial cell line (Figure 1D). Taken together, our results indicate that expression of LncRNA DANCR increases in prostate cancer.

### Knockdown of DANCR decreases migration and invasion of prostate cancer cells

**Figure 2:**
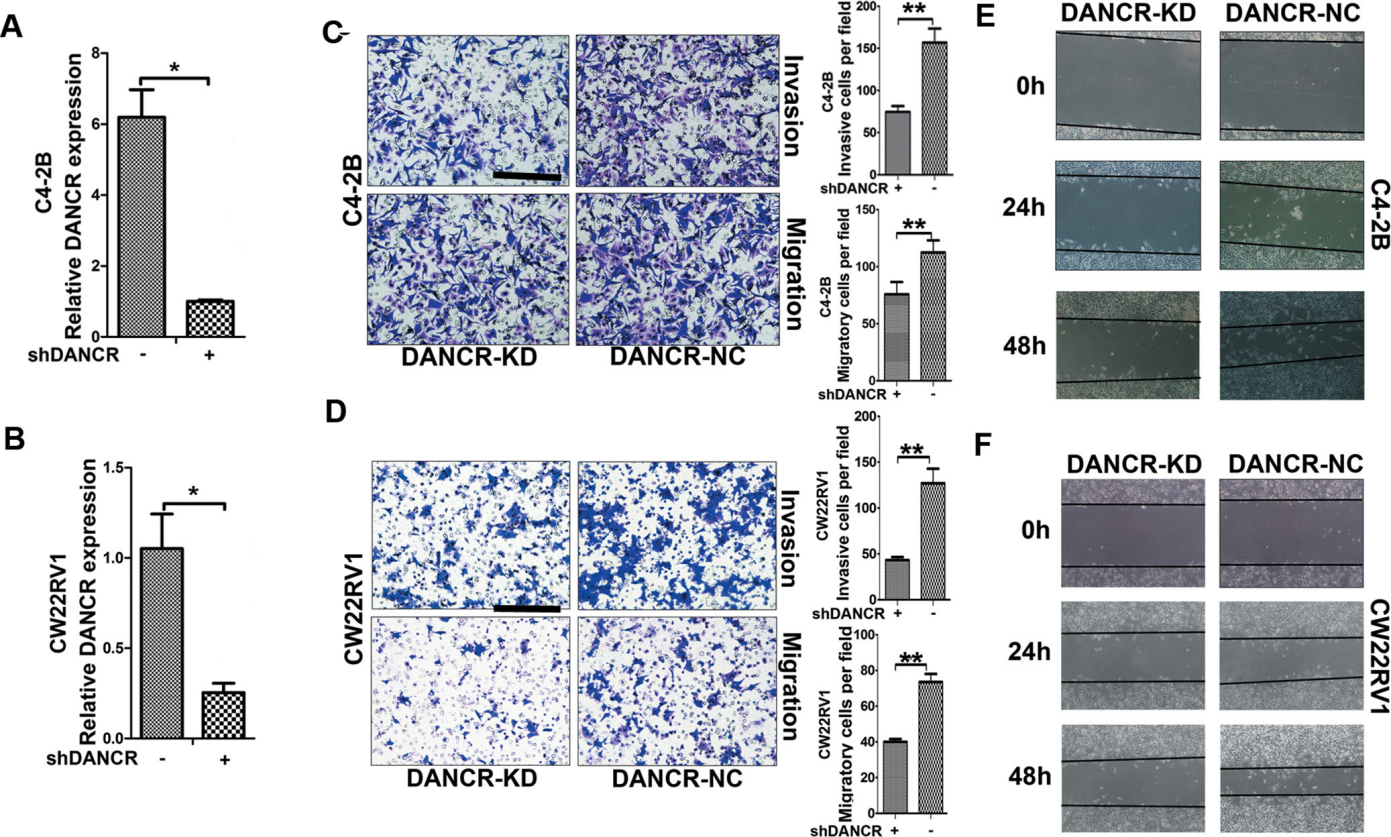
Knockdown of DANCR decreases migration and invasion of prostate cancer cells (**A**, **B**) C4-2B or CW22RV1 were transfected with indicated shRNA. The relative expression of DANCR were examined by RT-qPCR and normalized to GAPDH expression. The result expressed as the mean-SD, *P* < 0.05. (**C**, **D**) Knockdown of DANCR decreases migration and invasion of C4-2B or CW22Rv1cells, as detected by transwell assay. (**E**, **F**) Knockdown of DANCR decreases cell migration of C4-2B or CW22RV1 cells, as detected by wound healing assay. These results show data from at least three independent experiments, expressed as the mean ± SD. Representative figure of each experiment are shown at left. *p < 0.05, **p < 0.01, ***p < 0.001.

To further determine the function of DANCR in prostate cancer, lentivirus containing short hairpin RNA (shRNA) against DANCR was infected into androgen- independent C4-2B and CW22Rv1 prostate cancer cells and empty vector-containing lentivirus was used as control. As shown in Figure 2A and 2B, expression of DANCR was obviously knocked down in C4-2B and CW22Rv1 cells as verified by RT-qPCR assay. Firstly we detected whether DANCR regulates invasion and migration of prostate cancer cells. As shown in Figure 2C and 2D, DANCR knockdown suppressed both cell invasion and cell migration in C4-2B and CW22Rv1 cells as detected by transwell migration and transwell invasion assays. Moreover, we found that DANCR knockdown reduced cell motility through wound healing assay (Figure 2E and 2F), while it had no effect on cell proliferation (Supplementary Figure 1). These results indicate that DANCR promotes migration and invasion of prostate cancer cells.

### DANCR represses TIMP2/3 expression by mediating the binding of EZH2 on their promoters

**Figure 3:**
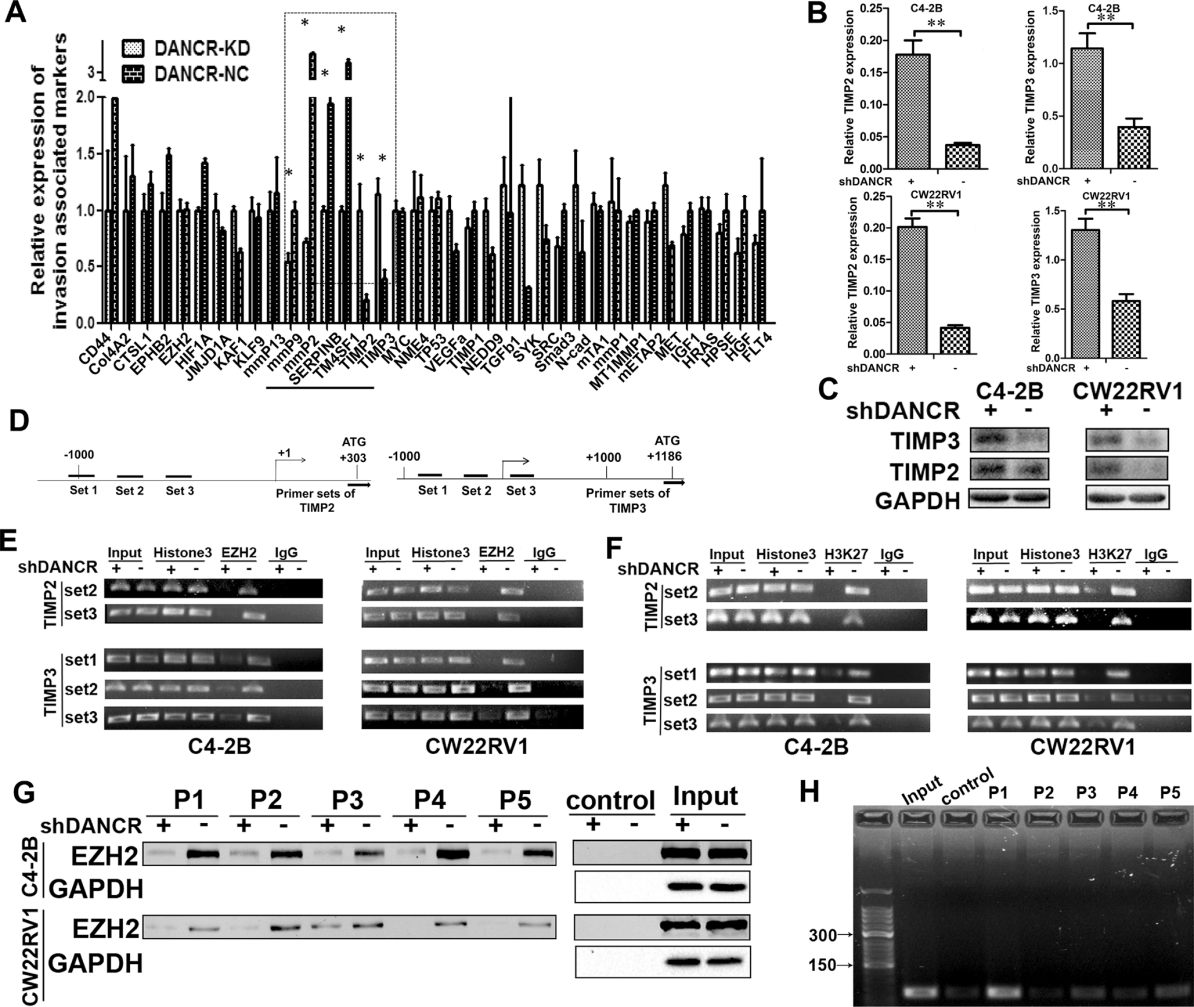
DANCR regulates TIMP2/3 expression by mediating the binding of EZH2 on its promoter (**A**) Effect of DANCR knockdown on the expression of thirty-seven invasion associated genes in C4-2B cells, as detected by RT-qPCR assay. (**B**, **C**) Knockdown of DANCR increases the expression of TIMP2/3 mRNA and protein in C4-2B and CW22RV1 cells, as detected by RT-qPCR assay and western blot analysis. (**D**) Representation of the TImP2/3 promoter region as mapped by PCR analysis and ChIP assay. The bent arrow represents the transcription start sites (+1). The lines below the TIMP2/3 locus represent the regions amplified by PCR. (**E**) Knockdown of DANCR decreased the binding of EZH2 on the promoter of TIMP2/3. Immuno-precipitated DNA was analyzed by PCR with specific primer sets. Chromatin obtained from C4-2B and CW22RV1 cells were immune-precipitated using antibodies to EZH2, histone H3 (H3) and IgG. Each ChIP experiment was repeated at least three times and a representative experiment is shown. (**F**) Knockdown of DANCR decreased the tri-methyl-histone H3K27 on the promoter of TIMP2/3. Immuno-precipitated DNA was analyzed by PCR with specific primer sets. Chromatin obtained from C4-2B and CW22RV1cells were immune-precipitated using antibodies to tri-methyl-histone H3K27 (3meH3K27), histone H3 (H3) and IgG. Each experiment was repeated at least three times and a representative experiment is shown. (**G**) Knockdown of DANCR decreased the binding of EZH2 on the promoter of TIMP2/3, as detected by oligonucleotides pull down assays. P1: TIMP2-set2; P2: TIMP2-set3; P3: TIMP3-set1; P4: TIMP3-set2; P5: TIMP3-set3. (**H**) DANCR was pulled down by the oligonucleotides with the same sequence of the promoters of TIMP2/3 (P1-P5). Control: The DNA solution TE buffer was used as negative control.

Since DANCR promoted cell invasion of C4- 2B and CW22RV1 cells, we further investigated the mechanism of how it regulates target genes. We examined the expression of 37 invasion related genes in DANCR knockdown C4-2B cells by RT-qPCR assay and found that DANCR knockdown significantly changed the mRNA level of MMP2, MMP9, TIMP2, TIMP3, SERPINB and TM4SF1 (Figure 3A). Considering the critical role of TIMP2/3 (TIMP metallopepitidase inhibitor 2/3) plays in prostate cancer invasion [15–16], we focused on how DANCR represses the expression of these two genes. As shown in Figure 3B and 3C, we further confirmed that both mRNA level and protein level of TIMP2/3 were significantly upregulated by DANCR knockdown in C4-2B cells and CW22Rv1 cells. These results suggested that DANCR maybe promote invasion in prostate cancer cells through down-regulation of TIMP2/3. Binding to Polycomb repressive complex 2 (PRC2) family member is an important way for LncRNAs to modulate the transcription of target genes [5–7]. Enhancer of zeste homolog 2 (EZH2) is the catalytic part of the PRC2, which catalyzes the trimethylation of Histone 3 on lysine 27 (H3K27me3) and induces chromatin compaction and transcription repression [17], and it was reported that DANCR could bind to EZH2 [18]. In addition, it was reported that EZH2 could bind to certain sets on TIMP2/3 promoter and inhibit their expression [19], and the schematic binding maps were showed in Figure 3D. Thus, we speculated that DANCR might regulate TIMP2/3 expression through modulating the binding of EZH2 on their promoter. To test this hypothesis, ChIP (Chromatin Immuno-precipitation) assay was applied and the primers to amplify these regions were designed. As shown in Figure 3E, DANCR knockdown decreased the binding of EZH2 on TIMP2/3 promoter in C4-2B and CW22Rv1 cells. Moreover, the binding of H3K27me3, which is a catalyzed product of EZH2, on TIMP2/3 promoter was also reduced by DANCR knockdown in C4- 2B cells and CW22Rv1 cells (Figure 3F). Moreover, we performed oligonucleotides pull down assay and further confirmed that EZH2 bound on the promoters of TIMP2/3 and the bindings were reduced by DANCR knockdown (Figure 3G). Interestingly, we found that DANCR was pulled down by the DNA fragments of TIMP2/3 promoter (Figure 3H), suggesting that DANCR also binds on promoters of TIMP2/3. Taken together, our results suggest that the binding of EZH2 and the methylation of H3K27me3 on TIMP2/3 promoter were decreased by DANCR knockdown, which may lead to the up-regulation of TIMP2/3, and DANCR promotes the binding of EZH2 on the promoter of TIMP2/3.

### The inhibition of TIMP2/3 by DANCR depends on EZH2

**Figure 4:**
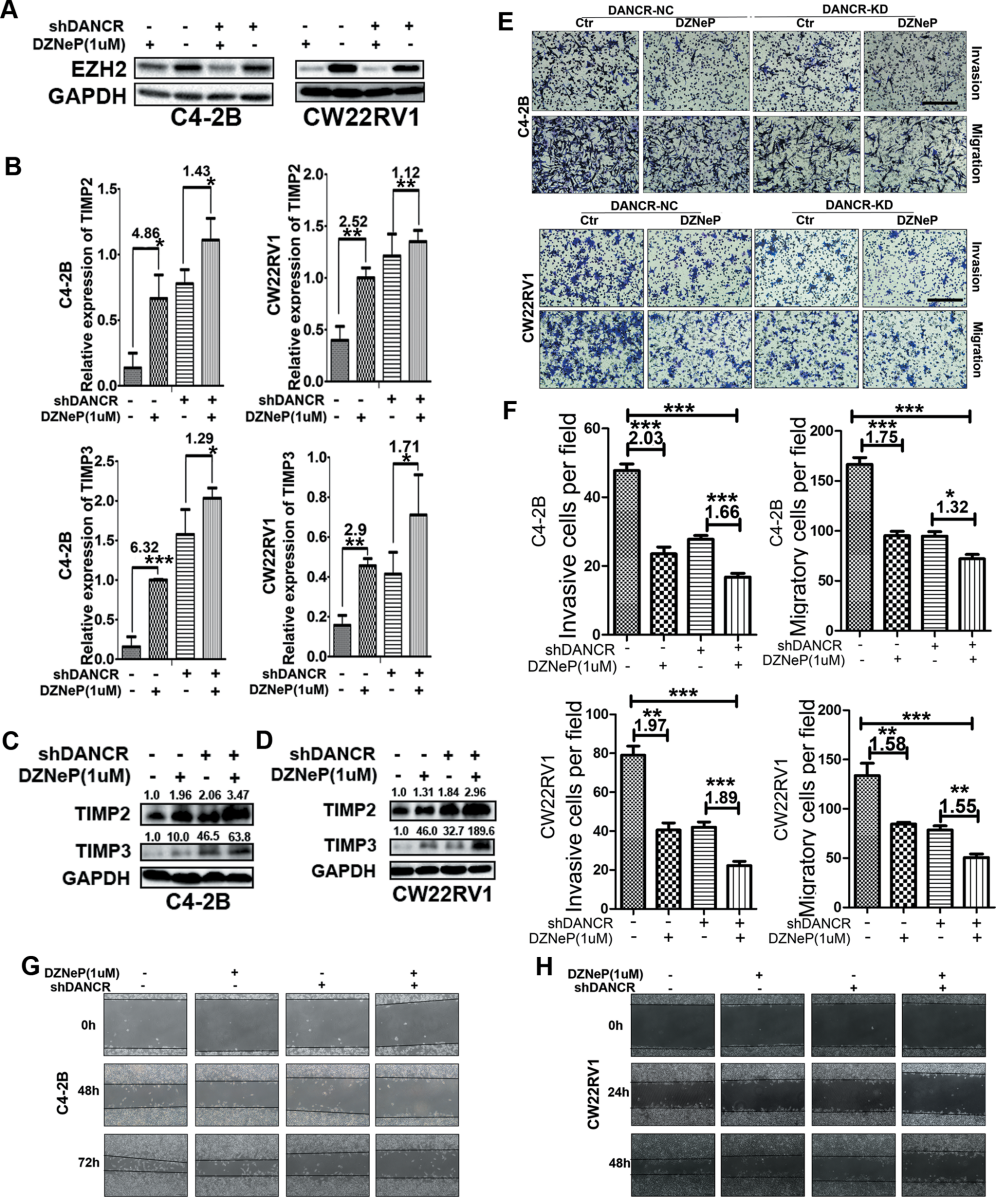
The inhibition of TIMP2/3 by DANCR depends on EZH2 (**A**) DZNep, an inhibitor of EZH2, decreases EZH2 protein level in both C4-2B and CW22Rv1 cells, as detected by western blot analysis. (**B**) Both DANCR knockdown and DZNep treatment increases the expression of TIMP2/3 mRNA in C4-2B and CW22Rv1 cells as detected by RT-qPCR assay. (**C, D**) Both DANCR knockdown and DZNep treatment increases the expression of TIMP2/3 protein in C4-2B and CW22Rv1 cells as detected by western blot analysis. (**E, F**) Both DANCR knockdown and EZH2 inhibition decreases invasion and migration of C4-2B and CW22Rv1 cells as detected by transwell assay. (**G**, **H**) Both DANCR knockdown and EZH2 inhibition decreases the migration of C4-2B and CW22Rv1 cells as detected by wound healing assay. Each experiment was repeated at least three times and a representative experiment is shown. *p < 0.05, **p < 0.01, ***p < 0.001.

To further confirm that EZH2 is necessary for DANCR to inhibit TIMP2/3, we used 3-Deaza-naplanocin A (DZNep), an inhibitor of EZH2, in C4-2B cells and CW22Rv1 cells and we detected the expression of TIMP2/3 and examined the invasive and migratory activity in DANCR knocked down cells and control cells. As shown in Figure 4A, treatment with 1μM of DZNep decreased EZH2 protein level while DANCR knockdown almost had no effect on EZH2 protein level. Next we detected the expression of TIMP2/3 at mRNA level (Figure 4B) and protein level (Figure 4C and 4D), and found the up-regulation of TIMP2/3 at both mRNA and protein level by DZNep treatment and DANCR knockdown separately and together, indicating that both EZH2 and DANCR could inhibit the expression of TIMP2/3. On the other hand, when cells were treated with DZNep, DANCR knockdown resulted less fold changes in the expression of TIMP2/3, for TIMP2 4.86 vs 1.43 and for TIMP3 6.32 vs 1.29 in C4-2B cells and for TIMP2 2.52 vs 1.12 and for TIMP3 2.90 vs 1.71 in CW22Rv1 cells respectively, suggesting that the inhibition of TIMP2/3 by DANCR depends on EZH2. The protein level changes of TIMP2/3 were similar with mRNA level changes in C4- 2B and CW22Rv1 cells (Figure 4C and 4D). As shown in Figure 4E and 4F, the invasive and migratory abilities were detected by transwell assay in these two cell lines, and less invasive and migratory abilities were detected in cells with DZNep treatment and DANCR knockdown separately and together. Wound healing assay results also showed that cell migration was decreased by DZNep treatment and DANCR knockdown (Figure 4G and 4H). In summary, these results indicate that EZH2 and DANCR synergistically regulate the transcription of TIMP2/3 and EZH2 is necessary for DANCR to repress the expression of TIMP2/3.

### Knockdown of DANCR decreases metastasis of CW22Rv1 prostate cancer cell xenograft in nude mice

**Figure 5:**
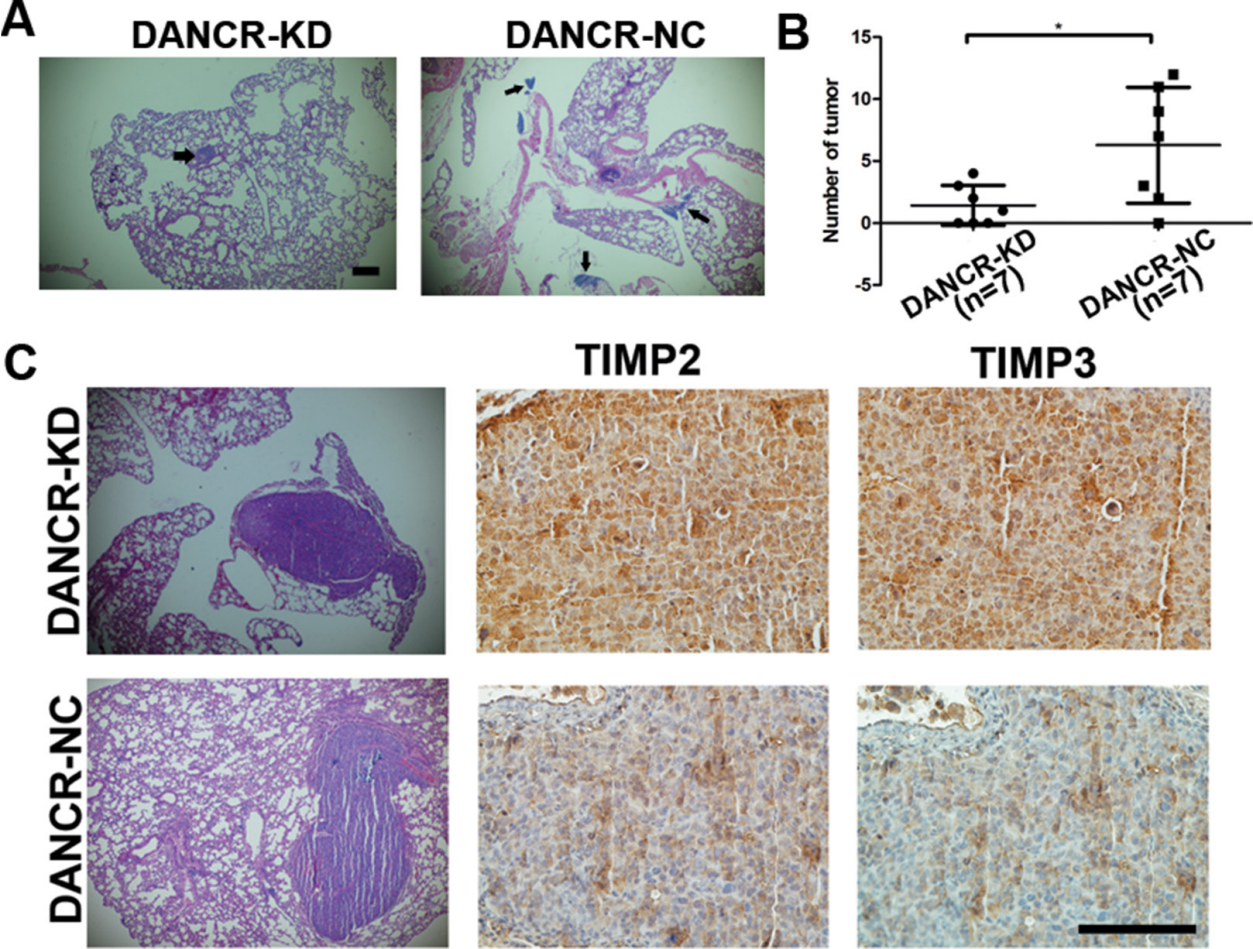
Knockdown of DANCR decreases migration and invasion of prostate cancer cells *in vivo* (**A, B**) Knockdown of DANCR decreased the number of metastatic foci of CW22Rv1 cells injected by tail vein in nude mice (7 mice in each group), as detected by histological analysis of mouse metastatic tumor tissue. The arrow indicates tumor foci in lung. (**C**) Knockdown of DANCR increases the expression of TIMP2/3 in the metastatic foci of CW22Rv1 cells injected by tail vein in nude mice, as detected by immunohistochemistry analysis of mouse metastatic tumor tissue. *p < 0.05.

To further determine whether DANCR promotes metastasis of prostate cancer, CW22Rv1-shDANCR or CW22Rv1-shNC cells were injected into nude mice by tail vein. More lung metastatic foci were detected in shNC group compared with the shDANCR group when mouse died or 8 weeks after injection by histological analysis (Figure 5A and 5B). Furthermore, we found that knockdown of DANCR increased the expression of TIMP2/3 in the metastatic foci of CW22Rv1-shDANCR compared with those of CW22Rv1-shNC, as detected by immunohistochemistry analysis of mouse metastatic tumor tissues (Figure 5C). These results are consistent with those of *in vitro* analysis, further confirming that DANCR represses the expression of TIMP2/3 and promotes the metastasis of prostate cancer cells.

### DANCR impedes the suppression of invasion and migration by androgen-AR signaling pathway in prostate cancer cells

**Figure 6:**
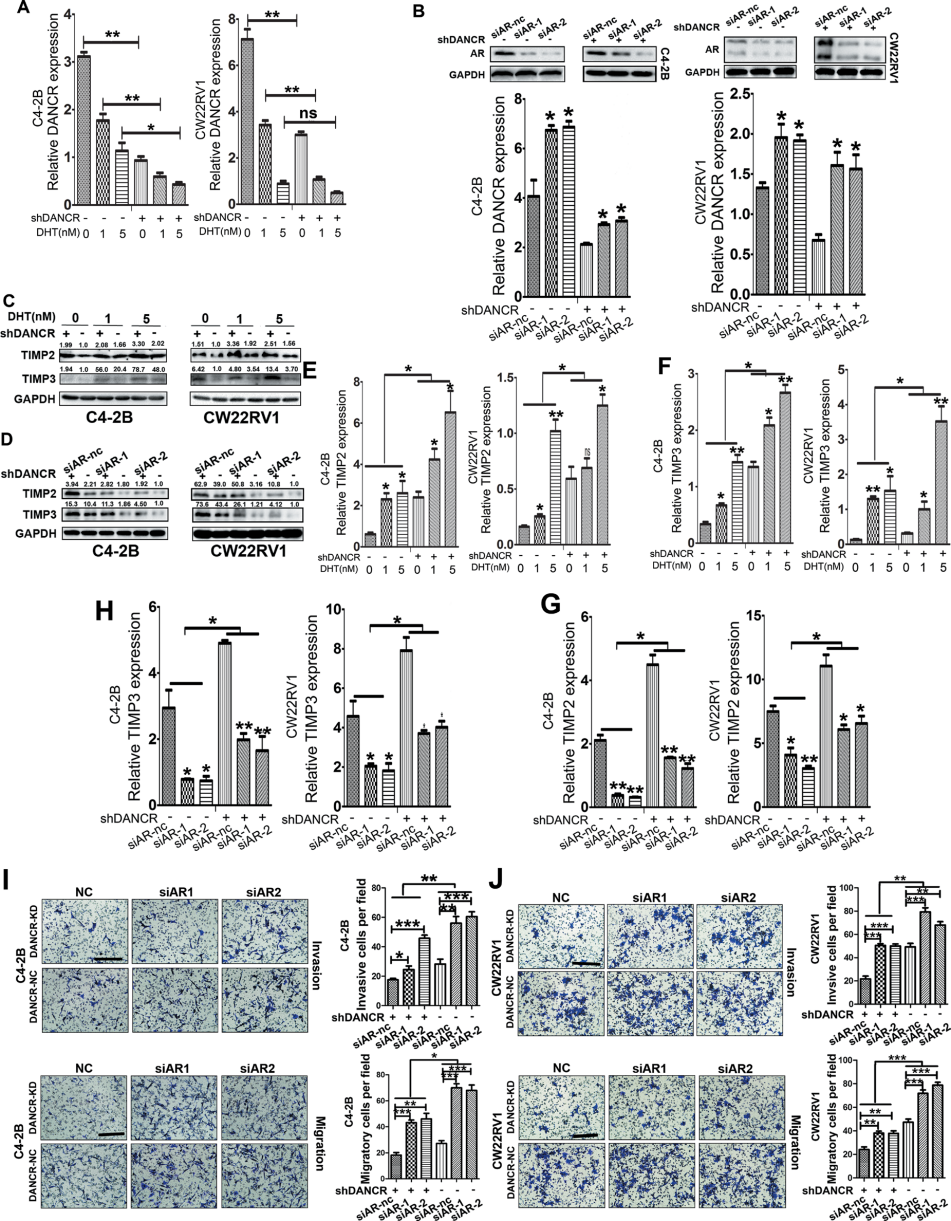
AR inhibits the expression of DANCR, up-regulates TIMP2/3 and suppresses invasion and migration of prostate cancer cells (**A**) Androgen inhibits the expression of DANCR in C4-2B and CW22RV1 cells, as detected by RT-qPCR assay. The mRNA level of DANCR were assayed after 24 hours of additional incubation with 1 nmol/L or 5 nmol/L DHT. (**B**) Knockdown of AR increases the expression of DANCR in C4-2B and CW22RV1 cells, as detected by RT-qPCR assay, and expression of AR was measured by western bot analysis. (**C**, **D**)Androgen treatment increases, while AR knockdown decreases the expression of TIMP2/3 protein in C4-2B and CW22RV1 cells as detected by western blot analysis. (**E**, **F**) Androgen treatment increases the expression of TIMP2/3 mRNA in C4-2B and CW22RV1 cells as detected by RT-qPCR assay. (**G**, **H**) AR knockdown decreases the expression of TIMP2/3 mRNA in C4-2B and CW22RV1 cells as detected by RT-qPCR assay. (**I, J**) Knockdown of AR increases the invasion and migration of C4-2B and CW22RV1 cells as detected by transwell assay. *p < 0.05, **p < 0.01, ***p < 0.001.

Androgen receptor (AR) plays a critical role in the development and treatment of prostate cancer. It was previously documented that AR is an inhibitor of prostate cancer metastasis [20–21]. To identify how AR regulates DANCR expression, we treated C4-2B and CW22Rv1 cells with DHT (dihydrotestosterone), and found that DANCR was downregulated by RT-qPCR assay (Figure 6A and Supplementary Figures 2–4) after AR was successfully activated, which was confirmed by upregulated mRNA level of PSA (Supplementary Figures 2–4). Furthermore, two small interfering RNA (si-AR) were used to knock down AR in these two cell lines and efficient AR knockdown was verified by western blot analysis and RT-qPCR assay (Figure 6B and Supplementary Figures 2–4), and we also found that DANCR was upregulated by AR knockdown through RT- qPCR assay. All these data suggested that DANCR was down regulated by androgen-AR signaling pathway in prostate cancer.

We further investigated whether DANCR affects AR function on the invasion of prostate cancer cells. Through western blot analysis and RT-qPCR assay, we detected TIMP2/3 expression at both protein level and mRNA level in C4-2B and CW22Rv1 cells with DANCR knockdown and DHT treatment, and found that TIMP2/3 were upregulated by DHT treatment and a higher basal mRNA level of TIMP2/3 in DANCR knockdown groups (Figure 6C, 6E and 6F, Supplementary Figures 2–4). Moreover, AR knockdown also downregulated TIMP2/3 expression at protein level and mRNA level (Figure 6D, 6G and 6H) Interestingly, DANCR knockdown promoted the up- regulation of TIMP2/3 by DHT treatment (Figure 6C, 6E and 6F) and decreased the down- regulation of TIMP2/3 by AR knockdown (Figure 6D, 6G and 6H). Our results indicate that androgen-AR signaling up-regulates TIMP2/3 and DANCR impedes this up-regulation in prostate cancer cells. Consistently, we examined the change of invasive and migratory abilities in C4-2B and CW22Rv1 cells with AR knockdown and DANCR knockdown, and found that AR knockdown promoted invasion and migration of prostate cancer cells and DANCR knockdown decreased the promotion of cell invasion and migration by AR down- regulation (Figure 6I and 6J). Collectively, our results suggest that AR up-regulates TIMP2/3, suppresses invasion and migration of prostate cancer cells and inhibits the expression of DANCR; moreover, DANCR impedes the upregulation of TIMP2/3 and the suppression of invasion and migration by androgen-AR signaling pathway.

### Enzalutamide treatment promotes invasion and migration of prostate cancer cells and DANCR knockdown decreased the promotion

**Figure 7:**
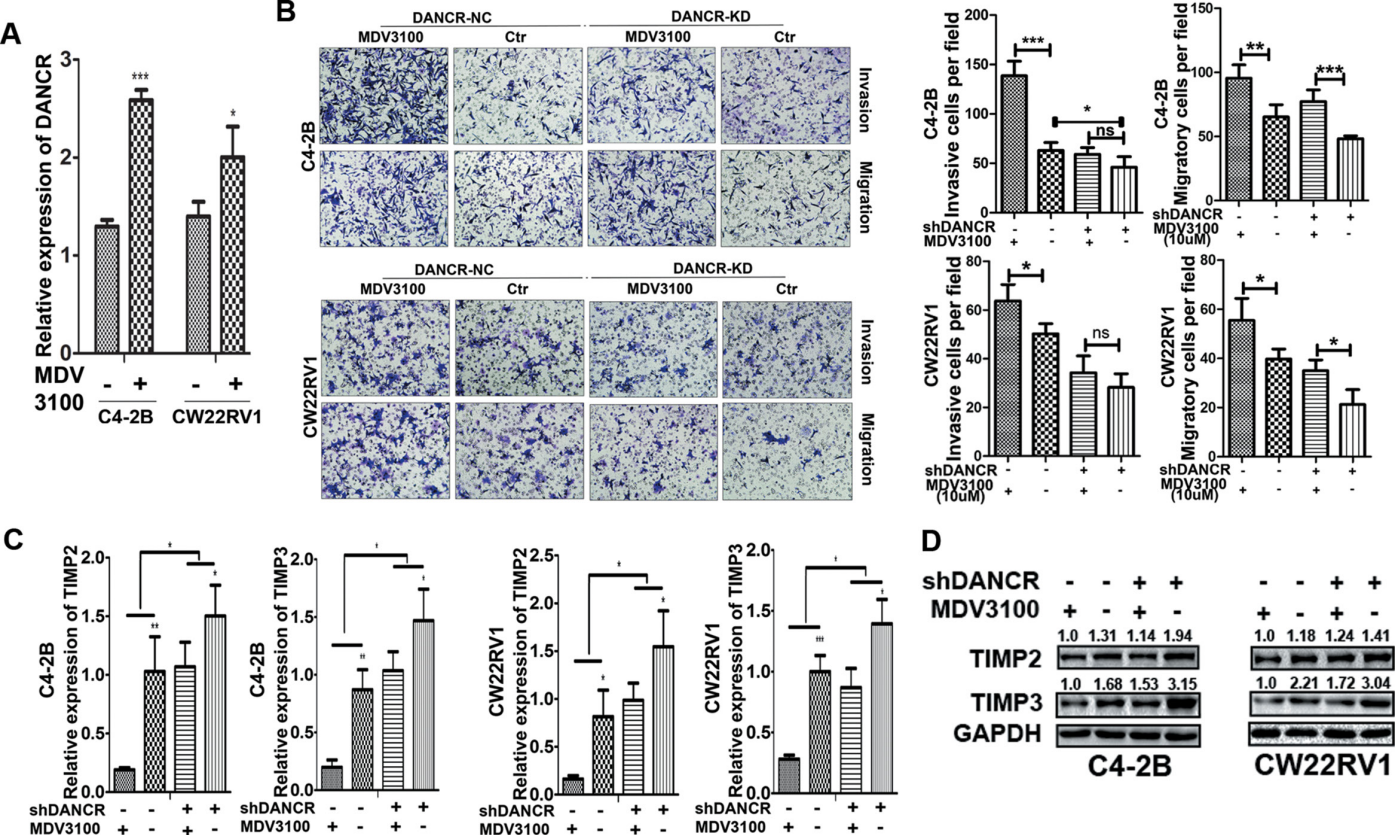
Enzalutamide treatment promotes invasion and migration of prostate cancer cells and DANCR knockdown decreased the promotion (**A**) Enzalutamide treatment increases the expression of DANCR in C4-2B and CW22RV1 cells, as detected by RT-qPCR assay. (**B**) Knockdown of DANCR decreases the cell invasion ability enhanced by enzalutamide in C4-2B and CW22RV1 cells, as detected by transwell assay. (**C**, **D**) Knockdown of DANCR increases expression of TIMP2/3 inhibited by enzalutamide in C4-2B and CW22RV1 cells, as detected by RT-qPCR assay and western blot analysis. *p < 0.05, **p < 0.01, ***p < 0.001.

Androgen deprivation therapy, which disrupts AR signaling through androgen ablation or AR antagonists, is the first-line treatment for disseminated prostate cancer. Enzalutamide (MDV 3100), an AR inhibitor, is widely used with prostate patients. However, it has been reported that enzalutamide promotes prostate cancer metastasis in different models [22, 23], moreover, in our study we found that AR knockdown promotes invasion and migration of prostate cancer cells while DANCR knockdown reduces the promotion. Therefore we want to determine whether DANCR knockdown could affect the effect of enzalutamide on prostate cancer cell invasion and migration. Firstly, we detected the expression of DANCR in C4-2B and CW22Rv1 cells treated with 5 μM of enzalutamide by RT-qPCR assay, and found that DANCR expression was increased by enzalutamide treatment (Figure 7A). Next we detected the cell invasion and migration ability in C4-2B and CW22Rv1 cells with enzalutamide treatment and DANCR knockdown and found that enzalutamide enhanced the cell invasion ability while DANCR knockdown decreased the enhancement (Figure 7B), moreover, expression of TIMP2/3 was reduced by enzalutamide at both mRNA and protein levels, and DANCR knockdown decreased the reduction (Figure 7C and 7D). Taken together, our results indicate that enzalutamide treatment promotes invasion and migration of prostate cancer cells and DANCR knockdown decreases the promotion, and suggest that DANCR knockdown may be useful for prevention of the potential side effect of AR inhibitors in prostate cancer treatment.

## DISCUSSION

In the present study, we found that lncRNA DANCR expression increased in prostate cancer, moreover, DANCR promoted invasion and migration of prostate cancer cells *in vitro* and enhanced metastasis of xenograft prostate tumor in mouse model. Mechanically, we found that TIMP2/3 are target genes of DANCR and knockdown of DANCR leads to up-regulation of TIMP2/3, decreased binding of EZH2 and H3K27me3 on the promoter of *TIMP2/3*. Furthermore, we confirmed that DANCR repressed expression of TIMP2/3 synergistically with EZH2. In summary, our results indicate that DANCR promotes invasion and metastasis of prostate cancer, and DANCR could be a potential target for the treatment of prostate cancer.

Expression of EZH2, which functions as an epigenetic gene silencer, increases in prostate cancer and it plays an oncogenic role in prostate cancer that is typically associated with increased risk of metastasis and recurrence [24–27]. In the present study, we found that DANCR knockdown leads to decreased binding of EZH2 on *TIMP2/3* promoter, indicating that DANCR may mediate the binding of EZH2 on them. On the other hand, we also found that DANCR and EZH2 synergistically repressed the expression of TIMP2/3, suggesting that the epigenetically silencing by EZH2 could play an essential role in the repression of TIMP2/3 expression by DANCR. Notably, it is possible that DANCR may activate EZH2 beyond its enzymatic activity since lncRNA can function as a scaffold to bind and bring transcription regulators to the promoter region of the target genes [5], and EZH2 can not only catalyze the methylation of histone 3, but also interact with other epigenetics regulator, such as DNMT1 and HDACs, and even transcription factors, and promotes the recruitment of them to the promoter region [28–29]. Thus, DANCR and EZH2 may form a larger complex with other transcription regulators and bring them to the promoter of TIMP2/3 to repress their transcription. Because EZH2 is an important target in prostate cancer [27], our results suggest that knockdown of DANCR could strengthen the effect of EZH2 inhibitor on the suppression of prostate cancer metastasis.

Androgen-AR signaling pathway is crucial in driving the terminal differentiation of prostate epithelial cells [30–31]. Interestingly, DANCR exerts a de-differentiation function in epidermis [12], therefore, we assumed that DANCR may antagonize the function of androgen-AR in prostate epithelial cells. Given that DANCR promotes the invasion (Figures 1–5) while AR inhibits the invasion of prostate cancer cells, we hypothesized that DANCR may antagonize the effect of androgen-AR on invasion and migration of prostate cancer cells. In the present study, we found that the migration and invasion of prostate cancer cells were suppressed by androgen-AR signaling while increased by enzalutamide, an inhibitor of AR, which is consistent with previous reports [32, 20]. Moreover, androgen-AR inhibited the expression of DANCR and increased TIMP2/3 expression. On the other hand, DANCR knockdown facilitated the upregulation of TIMP2/3 and the suppression of invasion and migration by androgen-AR (Figure 6), while DANCR knockdown decreased the promotion of invasion and migration in prostate cancer cells by enzalutamide treatment (Figure 7). Taken together, our results support that AR could inhibit some metastasis-promoting pathways or molecules in prostate cancer, and suggest that knockdown of DANCR could lessen the side effect of AR inhibitor, which may be useful in the treatment of prostate cancer.

Recent studies, utilizing specific cell surface markers and genetic lineage tracing approaches, have produced direct evidence that prostate stem cells are most likely AR-negative and it seems that androgen-AR pathway suppresses the stem cell phenotype and promotes cell differentiation [33–34]. Considering that DANCR increases stemness of epithelial cells [13], we deduce that DANCR may enrich in the basal layer of prostate and antagonizes the differentiation of prostate epithelial cells, on the other hand, it expression could be suppressed by AR in luminal cells. Whether the suppression of DANCR by AR plays an important role prostate epithelial cell differentiation is an interesting issue for us to cast light on in the future.

Although DANCR expression elevates in prostate cancer, the clinical relevance between DANCR and prostate cancer is still not clear. More human specimens are needed to clarify whether DANCR can be used as diagnostic and prognostic marker of prostate cancer. Moreover, several lncRNAs, such as PCA3 and MALAT-1, can be used as diagnostic urinary biomarkers for prostate cancer [35–36], thus DANCR expression in the urine of prostate cancer patients should be measured and analyzed in the future.

In conclusion, our results indicate that lncRNA DANCR promotes invasion and migration of prostate cancer cells *in vitro* and metastasis of tumor xenografts in nude mice, and decreases expression of TIMP2/3 synergistically with EZH2 through epigenetically silencing their promoter; moreover, DANCR expression is repressed by androgen-AR signaling pathway and DANCR knockdown facilitates the upregulation of TIMP2/3 and the suppression of invasion and migration by androgen-AR, while DANCR knockdown decreased the promotion of invasion and migration in prostate cancer cells by enzalutamide treatment. Our results suggest that DANCR could be a potential target for preventing prostate cancer metastasis, and knockdown DANCR may lessen the potential side effect of AR inhibitor.

## MATERIALS AND METHODS

### Cell lines and reagents

Human prostate cancer cell lines CWR22Rv1 and PC-3 were purchased from American Type Culture Collection (Manassas, VA, USA). C4-2B cell lines were a gift from Dr. Jer-Tsong Hsieh of University of Southwestern Medical Center. The three cell lines are all cultured in RPMI1640 medium supplemented with 10% fetal bovine serum (Gibco, NY, USA) at 37°C, in humidified air containing 5% of CO_2_. DNZep and enzalutamide (MDV3100) were purchased from Selleck Chemicals (Houston, TX, USA). DHT (dihydrotestosterone) were purchased from Sigma-Aldrich (St. Louis, MO, USA). All the reagents were reconstituted and stored following the protocol.

### Tissue acquisition and laser-capture microdissection

Human prostate cancer specimens were obtained from the Health Sciences Tissue Bank at the University of Pittsburgh Medical Center under approval by the UPMC Institutional Review Board following a standard protocol. All the specimens were from patients with prostate cancer with Gleason score 7 or above. The tissue process for laser-capture microdissection and mRNA level detection procedure were described previously [37, 38]. A detailed description of the pathological diagnosis for the patient samples in this study is provided in Supplementary Table 1.

### Western blot analysis

Cells were washed once with cold PBS and lysed in RIPA buffer (50 mM Tris pH 8.0, 150 mM NaCl, 0.1% SDS, 1% NP-40, and 0.5% sodium deoxycholate) containing protease inhibitors. Approximate 30 μg of protein was separated with 8–12% SDS-PAGE gel and blotted onto nitrocellulose membranes. Then membranes were blocked with 5% skim milk at room temperature for 1 hour and then incubated with primary antibodies against GAPDH (Kangchen, Shanghai, China), TIMP2, TIMP3 and androgen receptor (AR) (Santa Cruz, Dallas, TX, USA) at 4°C overnight, followed by TBST wash and 1 hour incubation with horseradish peroxidase-conjugated secondary antibodies at room temperature. Protein bands were visualized by a Molecular Imager ChemiDoc XRS System (Bio-Rad Laboratories, Hercules, CA, USA).

### Real time quantitive PCR (RT-qPCR) assay

Cells were harvested with Trizol reagent (Life Technologies, Rockville, MD, USA) to extract total RNA, which was reverse-transcribed to cDNA using PrimeScript™ RT reagent kit (Takara, Dalian, China). Then the cDNA was studied using CFX96 real-time PCR system (Bio-Rad, Hercules, CA) using SYBR Green PCR Master Mix (Takara, Dalian, China) to determine the transcriptional expression of specific genes. GAPDH was used for normalization. Relative gene expression was calculated by the 2^−ΔΔCt^ method. The primer sequences were given as followings: 18S, (F: 5′-GC AATTATTCCCCATGAACG-3′, R:5′-GGCCTCACTAAA CCATCCAA-3′); DANCR, (F:5′-GCCACTATGTAGAG GGTTTC-3′, R:5′-ACCTGCGCTAAGAA-CTGAGG-3′); PSA, (F:5′-GTGTGTGGACCTCCATGTTATT-3′, R:5′-CC ACT-CACCTTTCCCCTCAAG-3′), TIMP2, (F:5′-CTGG ACGTTGGAGGAAAGAA-3′, R:5′-GTCGAGAAACTCC TGCTTGG-3′), TIMP3, (F:5′-CTACCTGCCTTGCTTT-GTGAC-3′, R:5′-ATCCTCGGTACCAGCTGCAG-3′), GAPDH, (F:5′-ATGGGGA-AGGTGAAGGTCGG-3′, R:5′-GACGGTGCCATGGAATTTGC-3′).

### ShRNA and siRNA transfection

LV5 lentiviral vectors encoding short hairpin RNA (shRNA) targeting non-specific control (NC) or human Lnc RNA DANCR (5′- GGAGCTAGAGCAGTGACAATG -3′) were constructed by GenPharma (Shanghai, China). The siRNAs targeting Lnc RNA DANCR and non-specific control (NC) were purchased from RiboBio (Guangzhou, China). Cells achieved 70–80% confluence for lentiviral transfection or 30–50% confluence for siRNA transfection, and were transfected with X-tremeGENE siRNA Transfection Reagent (Roche, Germany) for 2–3 days, and harvested for the next experiments.

### Cell migration and invasion assay

Transwell migration and invasion assays were performed in 8-μm-pore transwell inserts (Millipore, Bedford, MA, USA). For *in vitro* invasion assays, the upper chambers of transwell were pre-coated with diluted matrigel (BD Biosciences, Sparks, MD), while for *in vitro* migration assays, no matrigel was used. 10^5^ × cells were seeded onto upper chamber in serum free medium and medium containing 10% serum were added to the lower chamber as a chemoattractant. After incubation for 24 h, the upper surface of the insert was wiped with a cotton swab and cells that migrated to the lower surface were fixed by 4% paraformaldehyde and stained with crystal violet. Cell numbers were counted in 6 random fields (200 ×) per well. Quantitation indicates mean ± SEM of triplicate repeats.

### Wound healing assay

Prostate cancer C4-2B or CW22RV1 cells with stably knocked down DANCR were seeded onto six-well plates. When the cell density reached above 90%, scratch wounds were made by scraping the cell layer in each culture plate using the tip of a 200-μl pipette. Then the cells were washed with PBS and cultured in serum-free medium with different treatment times, and then five fields (×100) were randomly chosen from each scratch wound and visualized by microscopy to evaluate the ability of cell migration. The experiments were performed in triplicates.

### Chromatin immunoprecipitation (ChIP) assay

ChIP assays were performed using SimpleChIP^®^ Enzymatic Chromatin IP Kit (Magnetic Beads) from Cell Signaling Technology (Danvers, MA, USA) following the manufacturer's protocol. Antibodies against EZH2 (CST), H3K27me3 (Abcam, Cambridge, UK) or normal rabbit IgG were used to precipitate protein/DNA complex. Precipitated DNA was analyzed by PCR with region-specific primers [19]: TIMP2-set2 (F:5′- TCATATGCCTGGGTCTTTCC-3′, R:5′-GGGGGTGTGGTTACTGTGAA-3′), TIMP2 -set3 (F:5′-GTTTCTCAATAGGCCACCCG-3′, R:5′-TTCCCCT TCAGCT-CGACTCT-3′), TIMP3-set1 (F:5′-TCTTCGG CCTCTGCTGTCCCA-3′, R:5′-GGTG-GCCAGCCAGG AACTCG-3′), TIMP3-set2 (F:5′-ACAGGATGAAGCGG AAGA-GA-3′, R:5′-TGTGGGTAGGAAAAGCAAGC-3′), TIMP3-set3 (F:5′-CGGGGGCA-AGGGCTTTGTGT-3′, R:5′-TGGTGGAACCAGCGGGGGAA-3′).

### Oligonucleotides pull-down assays

Oligonucleotides of the specific-region sets of TIMP2 and TIMP3, with biotin labeled on the 5′-end, were synthesized by GENEWIZ (Suzhou, China). The oligonucleotide sequences were as followings: TIMP2-set2 (F: biotin-5′-TCATATGCCTGGGTC- TTTCC-3′; R: biotin-5′-GGGGGTGTGGTTACTGTGAA-3′), TIMP2-set3 (F: biotin-5′-GTTTCTCAATAGGCCACCCG-3′; R:biotin-5′-TT CCCCTTCAGCTCGACTCT-3′), TIMP3-set1 (F: biotin-5′-TCTTCGGCCTCTGCTGTCCCA-3′; R: biotin-5′-GG TGGCCAGCCAGGAACTCG-3′), TIMP3-set2 (F: biotin-5′-ACAGGATGAAG- CGGAAGAGA-3′; R: biotin-5′-TG TGGGTAGGAAAAGCAAGC-3′), TIMP3-set3 (biotin-5′-CGGGGGCAAGGGCTTTGTGT-3′; R: biotin-5′-TGG TGGAACCAGC-GGGGGAA-3′). Each DNA fragment was amplified by PCR and purified with standard protocol. DANCR was knocked down in prostate cancer cells before cells were lysed. Procedures for pulling down DNA-bound proteins were described previously [39–40]. Finally, the EZH2 protein was detected by western blot analysis.

### Oligonucleotides pull-down RNA assays

Prostate cancer cells were lysed with RIPA buffer containing RNase inhibitor (CWBio, Beijing China) and cell lysates was incubated with biotin-labeling oligonucleotides for the specific-region sets of TIMP2 and TIMP3 overnight at 4°C. Then the biotin-oligonucleotides-protein-RNA complexes were pulled down with ImmunoPure streptavidin-agarose beads (20 μl/sample, Pierce, Rockford, IL, USA) and the complexes were freed from streptavidin-agarose beads by using 0.1 M biotin. The solution containing the complexed was treated with DNase I (CWBio) and Proteinase K (CWBio) before the RNA extracted with Trizol reagent, and then the RNA was reverse-transcribed to cDNA and PCR was performed to detect DANCR, and the primer sequences are as followings: F:5′-GCCACTATGTAGAGGGTTTC-3′, R:5′-AC-CTGCGCTAAGAACTGAGG-3′.

### Xenograft tumor model and *in vivo* metastasis analysis

CWR22Rv1 cells were stably knocked down DANCR and 4 × 10^6^ CWR22Rv1-shDANCR or CWR22Rv1-shNC cells were injected into seven male, 5 weeks old nude mice through tail vein, and 8-weeks later, mice were sacrificed and the metastases were examined by H&E staining. All animal studies were performed under the supervision and guidelines of the Institutional Animal Care and Use Committee of the Medical School, Xi'an Jiaotong University (Permission Number: SCXK2014-0155, 5 March 2014).

### Immunohistochemistry assay

The Immunohistochemistry staining were performed with EnVisionTM System (DAKO, Carpinteria, CA, USA), and the slides were de-paraffinized, rehydrated, followed by 5 min antigen retrieval, 10 minutes of endogenous enzyme block, and incubated with primary antibody (anti-TIMP2 and anti-TIMP3, Santa Cruz) overnight at 4°C. Then the slides were incubated with EnVision-HRP secondary antibody for 1 hour and the signal were detected by diaminobenzidine (DAB), followed by hematoxylin counterstaining. Staining signals were photographed using an Olympus BX51 Microscope (Olympus, Tokyo, Japan).

### Statistical analysis

All the statistical analyses were performed by GraphPad Prism (vesion5.0) software, and Student's *t* test was used when two groups comparison. *P* < 0.05 was considered statistically significant.

## SUPPLEMENTARY MATERIALS FIGURES AND TABLE


