# Supplementary material for: Long noncoding RNA DANCR promotes invasion of prostate cancer through epigenetically silencing expression of TIMP2/3
Source: Oncotarget. 2016 May 13;7(25):37868–81. doi: 10.18632/oncotarget.9350 (PMC5122356; doi:10.18632/oncotarget.9350)
Supplement: Supplementary file 1 [file oncotarget-07-37868-s001.pdf]

Long noncoding RNA DANCR promotes invasion of prostate cancer through epigenetically silencing expression of TIMP2/3

Supplementary Materials

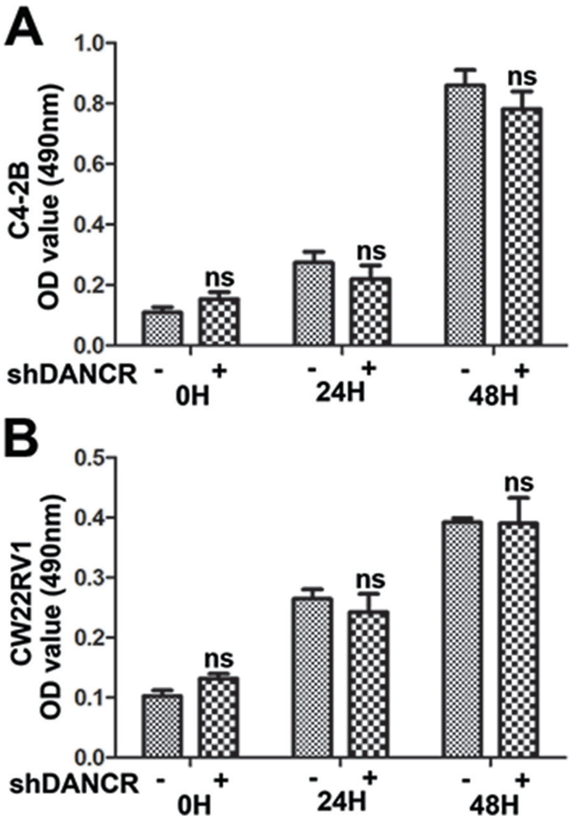

Supplementary Figure S1: DANCR knockdown does not affect prostate cancer cell proliferation. (**A, B**) Knockdown of DANCR does not change cell proliferation in C4-2B (A) and CW22Rv1 (B) cells, as detected by MTT assay.

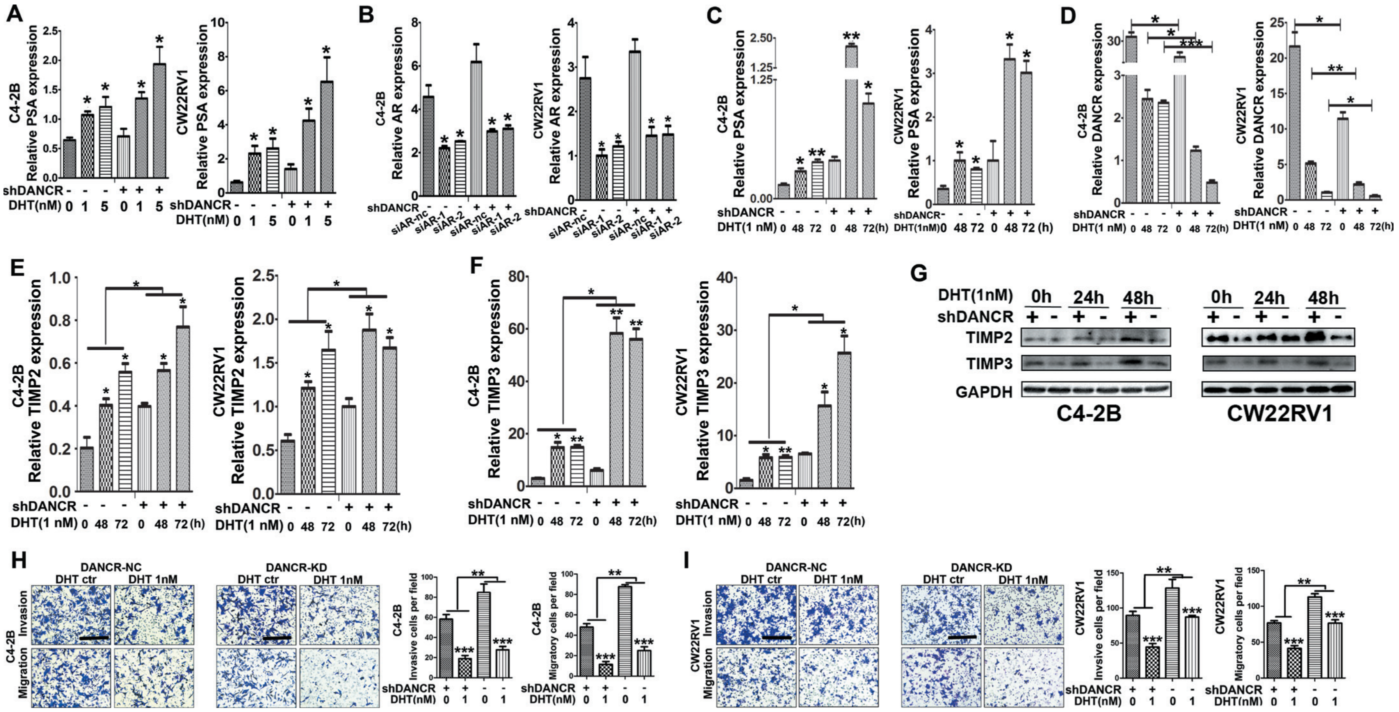

Supplementary Figure S2: (A) Androgen increases the expression of PSA mRNA in C4-2B and CW22RV1 cells. The mRNA level of PSA were assayed after 24 hours of additional incubation with 1 nM or 5 nM DHT. These results show data from at least three independent experiments, expressed as the mean SD, $p < 0.05$. (**B**) Expression of AR mRNA was decreased by transfection with indicated shRNA. (**C**) Androgen increases the expression of PSA mRNA in C4-2B and CW22RV1 cells. The mRNA level of PSA were assayed after additional incubation with 1 nmol/L DHT for 24 hrs or 48 hrs. (**D**) Androgen decreases the expression of DANCR at different treatment in C4-2B and CW22RV1 cells, as detected by RT-qPCR assay. (**E, F**) Androgen increases the expression of TIMP2/3 at different treatment in C4-2B and CW22RV1 cells, as detected by RT-qPCR assay. (**G**) Androgen increases the expression of TIMP2/3 with different treatment in C4-2B and CW22RV1 cells, as detected by western blot analysis. (**H, I**) Androgen suppresses the invasion and migration of C4-2B and CW22Rv1 cells as detected by transwell assay. These results show data from at least three independent experiments. *$p < 0.05$, **$p < 0.01$, ***$p < 0.001$.

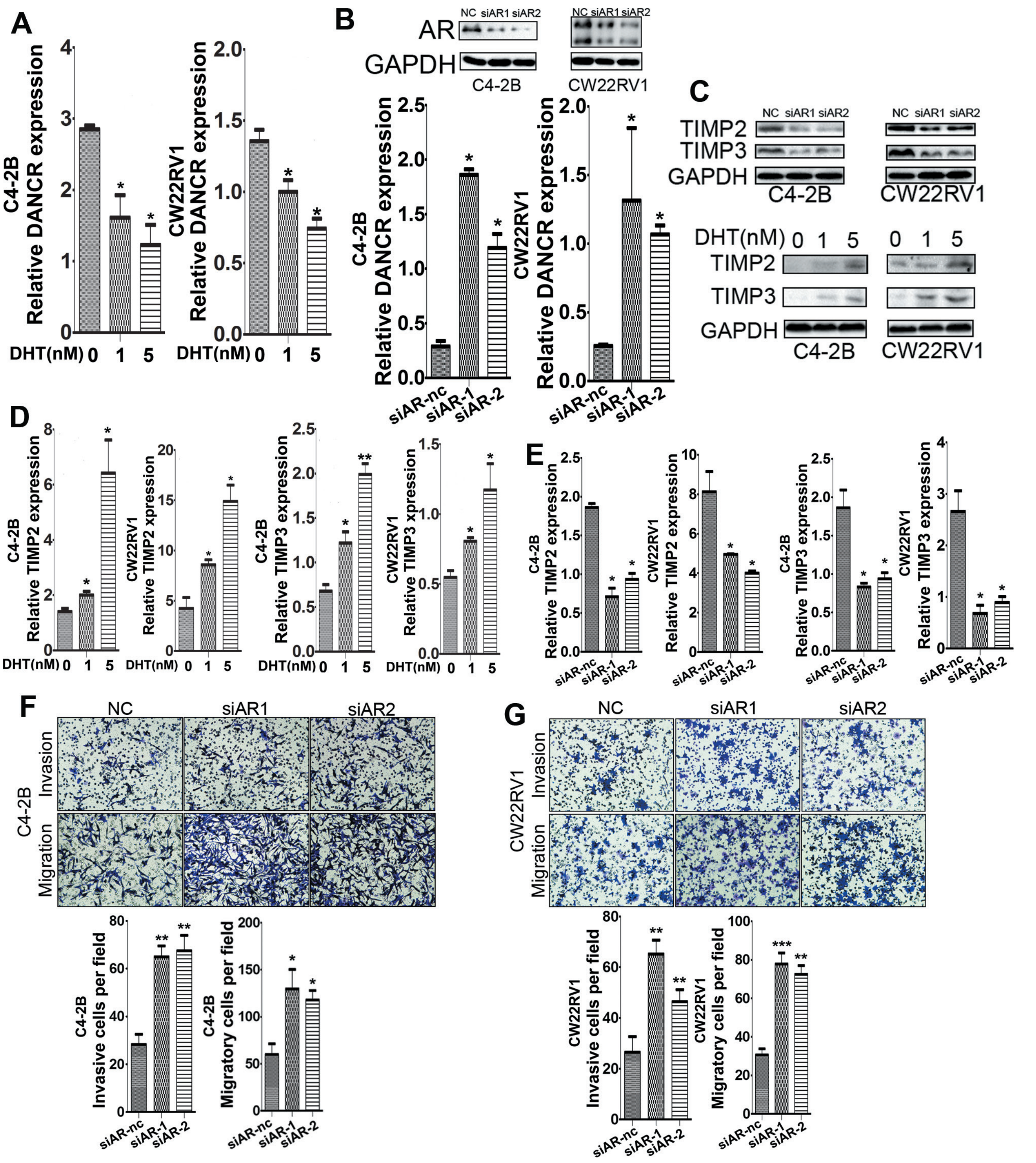

Supplementary Figure S3: Androgen-AR inhibits the expression of DANCR, up-regulates TIMP2/3 and suppresses invasion and migration of prostate cancer cells. (**A**) Androgen inhibits the expression of DANCR in C4-2B and CW22Rv1 cells, as detected by RT-qPCR assay. The mRNA level of DANCR were assayed after 24 hours of additional incubation with 1 nmol/L or 5 nmol/L DHT. (**B**) Knockdown of AR increases the expression of DANCR in C4-2B and CW22Rv1 cells, as detected by RT-qPCR assay, and expression of AR was measured by western bot analysis. (**C**) Androgen treatment increases, while AR knockdown decreases the expression of TIMP2/3 at protein level, as detected by western blot analysis. (**D**) Androgen treatment increases the expression of TIMP2/3 mRNA in C4-2B and CW22Rv1 cells, as detected by RT-qPCR assay. (**E**) AR knockdown decreases the expression of TIMP2/3 mRNA in C4- 2B and CW22RV1 cells, as detected by RT-qPCR assay. (**F, G**) Knockdown of AR increases the invasion and migration of C4-2B and CW22RV1 cells, as detected by transwell assay. These results show data from at least three independent experiments. $*p < 0.05$, $**p < 0.01$, $***p < 0.001$.

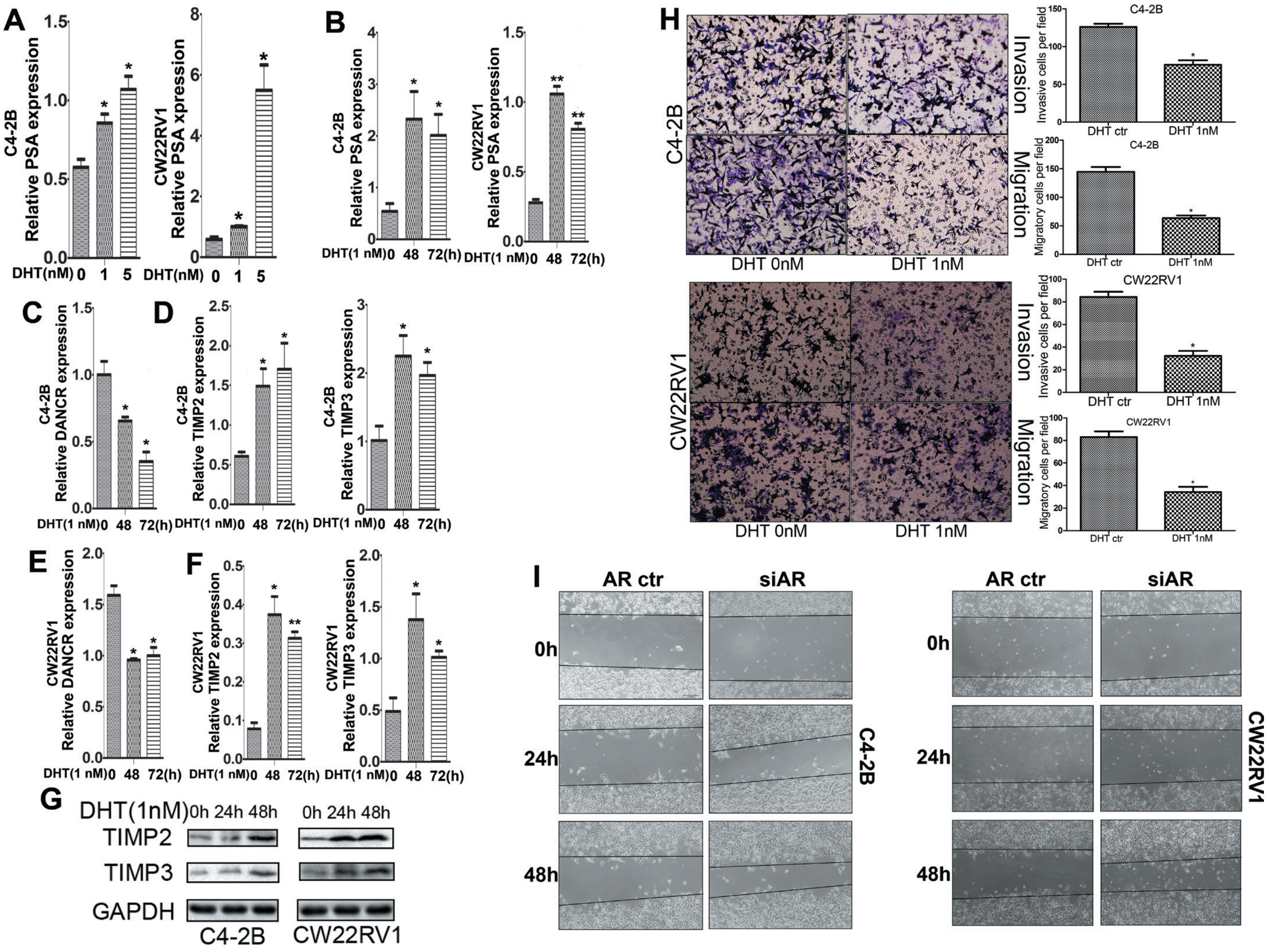

Supplementary Figure S4: Androgen-AR inhibits the expression of DANCR, up-regulates TIMP2/3 and suppresses invasion and migration of prostate cancer cells. (**A**) Androgen increases the expression of PSA mRNA in C4-2B and CW22Rv1 cells. The mRNA level of PSA were assayed after 24 hours of additional incubation with 1 nmol/L or 5 nmol/L DHT. These results show data from at least three independent experiments, expressed as the mean ± S.D., $p < 0.05$. (**B**) Androgen increases the expression of PSA mRNA in C4-2B and CW22Rv1 cells. The mRNA level of PSA were assayed after additional incubation with 1 nM DHT for 48 h or 72 h. (**C, E**) Androgen decreases the expression of DANCR at different treatment in C4-2B and CW22Rv1 cells, as detected by RT-qPCR assay. (**D, F**) Androgen increases the expression of TIMP2/3 at different treatment in C4-2B and CW22RV1 cells, as detected by RT-qPCR assay. (**G**) Androgen increases the expression of TIMP2/3 after additional incubation with 1 nM DHT for 48 h or 72 h in C4-2B and CW22Rv1 cells, as detected by western blot analysis. (**H**) Androgen decreases the cell invasion and migration ability as detected by transwell assay. (**I**) AR knockdown increases the migration of C4-2B and CW22Rv1 cells, as detected by wound healing assay. These results show data from at least three independent experiments. *$p < 0.05$, **$p < 0.01$, ***$p < 0.001$.

Supplementary Table S1: Patients' characteristics

**Prostate cancer (PCa)**	
Total number	12
Median age(range), y	50
**Sex**	
Male	12
**Stage**	
T1	0
pT2	3
pT3	8
pT4	1
**Gleason Score**	
6	0
7	4
≥ 8	8
**Operation**	
Radical prostatectomy	12
Radiation treatment	0
Hormone therapy	Unknown
